# Prevalence of contact allergy to metals in the European general population with a focus on nickel and piercings: The EDEN Fragrance Study

**DOI:** 10.1111/cod.12983

**Published:** 2018-04-10

**Authors:** Marie L. A. Schuttelaar, Robert F. Ofenloch, Magnus Bruze, Simone Cazzaniga, Peter Elsner, Margarida Gonçalo, Luigi Naldi, Åke Svensson, Thomas L. Diepgen

**Affiliations:** ^1^ Department of Dermatology University of Groningen, University Medical Center Groningen Groningen The Netherlands; ^2^ Department of Social Medicine, Occupational and Environmental Dermatology University Heidelberg Heidelberg Germany; ^3^ Department of Occupational and Environmental Dermatology Lund University, Skåne University Hospital Malmö Sweden; ^4^ Centro Studi GISED Bergamo Italy; ^5^ Dermatology Department Inselspital University Hospital Bern Switzerland; ^6^ Department of Dermatology University Hospital Jena Jena Germany; ^7^ Department of Dermatology, Faculty of Medicine Coimbra University Hospital, University of Coimbra Coimbra Portugal; ^8^ Department of Dermatology AULSS8 Berica, Ospedale San Bortolo Vicenza Italy; ^9^ Department of Dermatology Lund University, Skåne University Hospital Malmö Sweden

**Keywords:** chromium, cobalt, contact allergy, epidemiology, nickel, patch testing

## Abstract

**Background:**

Studies on sensitization to metals in the general population are scarce.

**Objectives:**

To determine the prevalence of sensitization to metals in the general population, and factors associated with nickel sensitization.

**Methods:**

In 5 European countries (The Netherlands, Germany, Italy, Portugal and Sweden), a random sample (*N* = 3119) from the general population (aged 18‐74 years) was patch tested and interviewed by use of a questionnaire on exposure to metals, piercing, and jewellery.

**Results:**

Overall, the age‐standardized prevalences of sensitization to nickel, cobalt and chromium were, respectively, 14.5%, 2.1%, and 0.8%. The highest prevalence of nickel sensitization was seen in Portugal (18.5%) and the lowest (8.3%) in Sweden. The prevalence of cobalt sensitization varied between 3.8% (The Netherlands) and 0.9% (Italy), and the prevalence of chromium sensitization varied between 1.3% (Portugal) and 0.2% (Sweden). Significant associations were observed between nickel allergy and female sex (odds ratio [OR] 5.19; 95% confidence interval [95%CI]: 3.99‐6.74), past piercing use (OR 3.86; 95%CI: 2.85‐5.24), and currently having ≥3 piercings (OR 5.58; 95%CI: 4.02‐7.76).

**Conclusions:**

The prevalence of sensitization to metals in the European general population was high, mostly because of nickel. The lowest prevalence of contact allergy to nickel and chromium observed in Sweden supports the effectiveness of long‐standing regulation.

## Graphical abstract

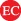

## 1 INTRODUCTION

Nickel, chromium and cobalt are patch tested in the European baseline series. Results on contact allergy to metals in consecutive dermatitis patients in Europe have been reported frequently,^1^, ^2^ whereas studies on sensitization to metals in the general population are scarce. Thyssen et al reported a median nickel allergy prevalence of 8.6% (range 0.7%‐27.8%) based on data mostly from western Europe and North America. The authors concluded that nickel was an important cause of contact allergy in the general population, and that contact allergy was widespread both among men and women, although it was more frequent in women.^3^ The objectives of our study were to estimate the prevalences of contact allergy to nickel, cobalt and chromium in the European general population, with a special focus on nickel allergy, exposure to piercings, and the risk of nickel sensitization.

## 2 MATERIALS AND METHODS

### 2.1 Study design

The study design and the data collection methods of the EDEN Fragrance Study have previously been published.^4^, ^5^ Briefly, the study was a descriptive epidemiological survey conducted in several European regions, including the metropolitan areas of Malmö (Sweden), Jena, Thüringen (Germany), Heidelberg, Baden‐Württemberg (Germany), and the provinces of Groningen (The Netherlands), Bergamo (Italy), and Coimbra (Portugal) in 2010. A random sample was selected from the general population, based on electoral precincts, aged 18 to 74 years.^4^ The contact information for each participant was gained from registers of residents in the corresponding regions. Before the participant was contacted, a random number was assigned to each dataset by the use of Microsoft Excel 2007. Participants were assigned to the patch test and non‐patch test groups according to this number. If a participant was enrolled in the patch test group, he or she was informed, after the interview had been performed. The refusal rate for the patch test after the interview was <1%. The study followed a stratified, proportional sampling‐with‐replacement design. Each potential participant was contacted by letter. Non‐responses were followed up with a second letter and a telephone call. If no contact was achieved, another individual was randomly selected in the same age and sex strata. The initial participation ranged from 20.3% to 50.7%, depending on the region.^4^ Non‐responder analysis was not performed. In total, 12 377 subjects were interviewed with a standardized questionnaire, of whom a random sample (*N* = 3119) was patch tested. The study was approved by the ethics committee of each participating centre.

### 2.2 Patch testing

The patch test procedure and the measures used to achieve a high degree of standardization have been previously published.^5^ The patch testing procedure was performed according to ICDRG/ESCD guidelines.^6^ The patch tests were applied on the back for 48 hours under occlusion, and readings were performed on day 3. Weak (+), strong (++) and extreme (+++) reactions with an allergic morphology were considered to be positive reactions. Reactions were considered to be irritant if margins were sharply demarcated and the surface of the test area showed a silk paper structure or a shiny skin. Reactions were considered to be doubtful if erythema and infiltration did not cover the whole test area. Metal contact allergy was defined as a positive patch test reaction (+/++/+++) to at least 1 of the following allergens: nickel sulfate, cobalt dichloride, or potassium dichromate. All patients were patch tested with the TRUE Test, which contains nickel sulfate (concentration 0.20 mg/cm^2^), cobalt dichloride (concentration 0.02 mg/cm^2^), and potassium dichromate (concentration 0.023 mg/cm^2^).

### 2.3 Data collection and analysis

As reported previously, the interview was conducted face‐to‐face with a trained interviewer, and consisted of three parts.^4^, ^5^ The interview comprised, among other things, demographic and personal characteristics, and a description of a previous diagnosis of atopic dermatitis made by a physician or dermatologist. A lifetime prevalence of hand eczema was registered as follows: the interviewer asked whether the participant had ever experienced an itchy skin rash that lasted for >3 days, and subsequently the location of this rash on the body. Hand eczema was defined as being present if the location was the hand, and a diagnosis of contact dermatitis, atopic dermatitis or other dermatitis was registered. In addition, a detailed history of exposure to metals, piercing and jewellery was taken.

All statistical analyses were performed with SPSS 23 (IBM, Armonk, New York). Descriptive data are presented in tables as numbers with percentages and 95% confidence intervals (95%CIs). The main information is also presented in strata of sex and country of origin. Prevalences are presented as both crude estimates and age‐standardized estimates with accompanying 95%CIs. Age standardization was performed according to the direct method.^7^ The European standard population was taken as the reference for standardization. Measures of association between nickel allergy and main variables are expressed as odds ratio (ORs) with 95%CIs. OR estimates were obtained by univariate logistic regression analysis in a first step. Then, a multivariate analysis including all variables that were significant in the univariate model was performed in order to control for potential confounders. ORs were considered to be statistically significant when 1 was not included in the 95%CI.

## 3 RESULTS

### 3.1 Socio‐demographic characteristics

The socio‐demographic characteristics of the 3119 patch tested subjects are shown in Table 1, together with the characteristics of the prevalence sample; these data have already been partly presented in Diepgen et al.^8^ It can be seen that the subsample patch tested was quite comparable to the prevalence sample of the general population. The percentage of females was slightly higher (54.9% females vs 45.1% males) in both samples. Age distribution did not differ substantially between the 2 samples, showing a slightly lower percentage of patch tested subjects in the oldest age group (71‐84 years). In the subsample patch tested, 51.7% of the subjects had ever had a piercing and 10% had currently ≥3 piercings; these numbers were also comparable to those in the prevalence sample. The prevalence of ever having a piercing was significantly higher in females (81.5%) than in males (14.9%; *P* < .01). The prevalence of currently having ≥3 piercings was also higher in females than in males (17.0% females vs 1.5% males).

**Table 1:** Sociodemographic characteristics of patch tested subjects

	Prevalence sample (*N* = 12 377)			Subsample patch tested (*n* = 3319)		
	*n*	%	95%CI	*n*	%	95%CI
Sex						
Male	5701	46.1	45.2‐47.0	1405	45.1	43.3‐46.8
Female	6669	53.9	53.0‐54.8	1712	54.9	53.2‐56.7
Age						
18 to 30 years	3498	28.3	27.5‐29.1	828	26.5	25.0‐28.1
31 to 45 years	3314	26.8	26.0‐27.6	824	26.4	24.9‐28.0
46 to 60 years	3150	25.5	24.7‐26.2	855	27.4	25.9‐29.0
61 to 74 years	2415	19.5	18.8‐20.2	612	19.6	18.2‐21.1
Atopic dermatitis in lifetime	883	7.8	7.3‐8.3	220	7.6	6.7‐8.6
Hand dermatitis in lifetime	358	3.9	3.5‐4.3	110	3.5	2.9‐4.2
Need to avoid metals/jewels ever	3732	30.2	29.3‐31.0	941	30.2	28.6‐31.8
Piercing ever	5987	49.4	48.5‐50.3	1563	51.7	49.9‐53.5
Currently ≥3 piercings	1258	10.4	9.8‐10.9	303	10.0	9.0‐11.2

### 3.2 Patch test reactions to nickel, cobalt, and chromium

Table 2 shows the crude prevalences of contact allergy, defined as at least 1 positive patch test reaction (+/++/+++), to nickel, cobalt and chromium separately, and to at least 1 of the 3 metals. In addition, a subdivision of the test results by sex and the age‐standardized prevalences in the different countries are shown. Overall, 489 of 3117 patch tested subjects reacted positively to at least 1 metal, yielding a crude prevalence of 15.7%, and an identical age‐standardized prevalence of 15.7%. The crude prevalences of contact allergy to nickel, cobalt and chromium were, respectively, 14.5%, 2.2%, and 0.8%, and the age‐standardized prevalences were, respectively, 14.5%, 2.1%, and 0.8%.

**Table 2:** Patch test results with metals stratified by country

	Males				Females				Crude prevalence				Age‐standardized prevalence			
	+/++/+++			Total	+/++/+++			Total	+/++/+++			Total	+/++/+++			Total
	*n*	%	95%CI	*n*	*n*	%	95%CI	*n*	*n*	%	95%CI	*n*	*n*	%	95%CI	*n*
Nickel sulfate																
Germany	23	4.9	3.1‐7.3	468	120	21.3	18.0‐24.9	564	142	13.9	11.8‐16.1	1024	143	13.9	11.8‐16.1	1032
Italy	13	6.3	3.4‐10.5	207	74	23.0	18.4‐27.9	323	95	17.4	14.3‐20.8	546	87	16.4	13.3‐19.8	531
The Netherlands	21	8.9	5.5‐13.0	241	56	22.6	17.5‐28.2	249	77	15.7	12.6‐19.3	489	78	15.8	12.8‐19.4	491
Portugal	10	4.3	2.1‐7.7	234	88	29.5	24.3‐35.0	299	97	18.3	15.1‐21.8	531	98	18.5	15.2‐21.9	533
Sweden	7	2.9	1.2‐5.8	244	35	13.4	9.6‐18.2	260	40	7.7	5.6‐10.4	518	42	8.3	6.1‐11.1	505
Total	74	5.3	4.2‐6.6	1395	374	22.0	20.1‐24.1	1696	451	14.5	13.3‐15.8	3108	448	14.5	13.3‐15.8	3091
Cobalt dichloride																
Germany	3	0.7	0.1‐1.9	468	20	3.6	2.2‐5.4	564	23	2.2	1.4‐3.4	1024	24	2.3	1.5‐3.4	1032
Italy	1	0.4	0‐2.7	207	4	1.3	0.3‐3.1	323	6	1.1	0.4‐2.4	546	5	0.9	0.3‐2.2	531
The Netherlands	6	2.4	0.9‐5.3	244	13	5.2	2.7‐8.5	256	19	3.8	2.3‐5.9	498	19	3.8	2.3‐5.9	500
Portugal	5	2.1	0.7‐4.9	234	8	2.7	1.2‐5.2	299	13	2.4	1.3‐4.2	531	13	2.5	1.3‐4.1	533
Sweden	0	0.0	0‐1.5	244	6	2.2	0.9‐5.0	260	7	1.4	0.5‐2.8	518	6	1.1	0.4‐2.6	505
Total	15	1.1	0.6‐1.8	1397	51	3.0	2.2‐3.9	1703	68	2.2	1.7‐2.8	3117	66	2.1	1.7‐2.7	3100
Potassium dichromate																
Germany	6	1.2	0.5‐2.8	468	6	1.1	0.4‐2.3	564	12	1.2	0.6‐2.0	1024	12	1.1	0.6‐2.0	1032
Italy	2	0.9	0.1‐3.4	207	0	0.0	0‐1.1	323	2	0.4	0‐1.3	546	2	0.4	0‐1.4	531
The Netherlands	1	0.5	0‐2.3	244	1	0.3	0‐2.2	256	2	0.4	0‐1.4	498	2	0.4	0‐1.4	500
Portugal	4	1.7	0.5‐4.3	234	3	1.0	0.2‐2.9	299	7	1.3	0.5‐2.7	531	7	1.3	0.5‐2.7	533
Sweden	1	0.2	0‐2.3	244	1	0.2	0‐2.1	260	2	0.4	0‐1.4	518	1	0.2	0‐1.1	505
Total	13	1.0	0.5‐1.6	1397	11	0.6	0.3‐1.2	1703	25	0.8	0.5‐1.2	3117	24	0.8	0.5‐1.1	3100
At least one positive reaction to a metal																
Germany	29	6.2	4.2‐8.8	468	130	23.0	19.6‐26.8	564	158	15.4	13.3‐17.8	1024	159	15.4	13.3‐17.8	1032
Italy	14	6.8	3.7‐11.1	207	76	23.4	19.0‐28.5	323	98	17.9	14.8‐21.4	546	90	16.9	13.9‐20.4	531
The Netherlands	26	10.7	7.1‐15.2	244	61	24.0	18.7‐29.5	256	86	17.3	14.1‐20.9	498	87	17.5	14.2‐21.0	500
Portugal	14	6.0	3.3‐9.8	234	92	30.9	25.6‐36.3	299	105	19.8	16.5‐23.4	531	106	20.0	16.6‐23.5	533
Sweden	8	3.1	1.4‐6.4	244	35	13.6	9.6‐18.2	260	42	8.1	5.9‐10.8	518	43	8.5	6.2‐11.3	505
Total	91	6.5	5.3‐7.9	1397	394	23.2	21.2‐25.2	1703	489	15.7	14.4‐17.0	3117	485	15.7	14.4‐17.0	3100

A subdivision by country showed that the highest age‐standardized prevalence of contact allergy to at least 1 metal was in Portugal (20.0%) and the lowest was in Sweden (8.5%); this was mostly attributable to nickel in both countries. Portugal (18.5%) had the highest age‐standardized prevalence of nickel allergy, followed by Italy (16.4%), The Netherlands (15.8%), and Germany (13.9%), whereas the Swedish prevalence (8.3%) was approximately half as high. Concerning cobalt, The Netherlands (3.8%) had the highest prevalence, whereas Sweden (1.1%) and Italy (0.9%) had the lowest age‐standardized prevalences. Regarding chromium, the highest prevalences were found for Portugal (1.3%) and Germany (1.1%), whereas low prevalences were found for Italy and The Netherlands (both 0.4%). The lowest age‐standardized prevalence of chromium contact allergy was found for Sweden (0.2%).

Concerning results stratified by sex, across all study centres, the prevalence of nickel contact allergy was much higher in females (22.0%) than in males (5.3%); the prevalence of cobalt contact allergy was also higher in females (3.0%) than in males (1.1%), whereas, for chromium, the prevalence in females (0.6%) was lower than in males (1.0%). The highest prevalence of nickel allergy in Portugal was found in females (29.5%) but not in males (4.3%). In males, the highest prevalence of nickel allergy was found in The Netherlands (8.9%), whereas the lowest prevalence was found in Sweden (2.9%). Regarding chromium, the subdivision by sex showed comparable prevalences in males and females in Germany, The Netherlands, and Sweden. In Italy, the prevalence of chromium allergy was higher in males (0.9%) than in females (0.0%); this was also observed in Portugal (males, 1.7%; females, 1.0%).

### 3.3 Patch test reactivity to nickel

The grades of patch test results of all patients who were positive for nickel, marked by country, are shown in Table 3. Strong (++) and extreme (+++) patch test reactions were combined as 1 group (++/+++). Overall, more strong/extreme positive reactions (10.3%) were observed than weak positive reactions (4.2%) to nickel. The distribution between strong/extreme and weak was not similar across the different countries. In Sweden, almost all sensitized subjects had strong/extreme positive reactions (8.2%) rather than weak positive reactions (0.1%). Also in Italy and Portugal, many more strong/extreme positive reactions (14.3%) were seen than weak positive reactions (1.1%). In Germany and The Netherlands, the differences between strong/extreme and weak reactions were not so obvious, being, respectively, 7.9% and 6.0% for Germany, and 9.1% and 6.7% for The Netherlands. The highest prevalence of irritant reactions was observed in Sweden (3.7%), and the lowest prevalence of irritant reactions was observed in Portugal (0%). The highest prevalence of doubtful reactions was observed in Germany (4.1%), and the lowest prevalence of doubtful reactions was observed in Italy (0.2%).

**Table 3:** Strength of positive reactions (+ vs ++/+++), ?+ and irritant reactions (IRs) to nickel stratified by country (age‐standardized)

		+		++/+++		?+		IR			
	*N* tested	*n*	%	*n*	%	*n*	%	*n*	%	% positive age‐standardized	95%CI
Germany	1032	62	6.0	82	7.9	42	4.1	10	1.0	13.9	11.8‐16.1
Italy	531	14	2.6	74	13.9	1	0.2	2	0.4	16.4	13.3‐19.8
The Netherlands	493	33	6.7	45	9.1	23	4.6	4	0.8	15.8	12.8‐19.4
Portugal	533	22	4.1	76	14.3	6	1.1	0	0	18.5	15.2‐21.9
Sweden	505	1	0.1	41	8.2	8	1.6	19	3.7	8.3	6.1‐11.1
Total	3093	131	4.2	317	10.3	80	2.6	35	1.1	14.5	13.3‐15.8

### 3.4 Nickel allergy stratified by sex and piercings

The prevalence of nickel allergy stratified by sex, age group and never having had a piercing, having ever had a piercing but not currently, currently having 1 to 2 piercings, and currently having ≥3 piercings, respectively, is shown in Table 4. Overall, subjects who ever had a piercing but did not currently have a piericing showed a higher percentage of nickel allergy than the group who never had a piercing, namely 20.8% and 6.4%, respectively. The frequency of nickel allergy increased with the number of current piercings. Subjects with ≥3 piercings currently showed the highest prevalence of nickel allergy (27.6%), followed by subjects with 1 to 2 piercings currently, who had a prevalence of nickel allergy of 21.4%. Subdivision into age groups showed that, in subjects with ≥3 piercings currently, high prevalences of nickel allergy were found in all age groups: respectively, 24.3% (18‐30 years), 30.4% (31‐45 years), 33.3% (46‐60 years), and 23.1% (61‐74 years). In females, the highest prevalence (31.8%) was seen in the group aged 31 to 45 years, whereas in males the highest prevalence (7.9%) was seen in the group aged 46 to 60 years. In females, after increasing with age, the prevalence decreased from the group aged 46 to 60 years. In males, after increasing with age, the prevalence decreased in the oldest group aged 61 to 74 years.

**Table 4:** Prevalence of nickel contact allergy stratified by age groups and piercings. CI, confidence interval

		*n* tested	*n* positive	% crude positive	95%CI
Total		3110	451	14.5	13.3‐15.8
	Males	1403	73	5.2	4.1‐6.5
	Females	1705	378	22.2	20.2‐24.2
	Never piercing	1457	93	6.4	5.2‐7.8
	Piercing ever but not currently	475	99	20.8	17.3‐24.8
	Currently 1 to 2 piercings	780	167	21.4	18.6‐24.5
	Currently ≥3 piercings	301	83	27.6	22.6‐33.0
18 to 30 years	Males	386	13	3.4	1.8‐5.7
	Females	439	87	19.8	16.2‐23.9
	Never piercing	329	12	3.6	1.9‐6.3
	Piercing ever but not currently	118	17	14.4	8.6‐22.1
	Currently 1 to 2 piercings	213	35	16.4	11.7‐22.1
	Currently ≥3 piercings	148	36	24.3	17.7‐32.1
31 to 45 years	Males	359	22	6.1	3.9‐9.1
	Females	462	147	31.8	27.6‐36.3
	Never piercing	349	26	7.4	4.9‐10.7
	Piercing ever but not currently	153	46	30.1	22.9‐38.0
	Currently 1 to 2 piercings	206	65	31.6	25.3‐38.4
	Currently ≥3 piercings	92	28	30.4	21.3‐40.9
46 to 60 years	Males	354	28	7.9	5.3‐11.2
	Females	496	109	22.0	18.4‐25.9
	Never piercing	417	43	10.3	7.6‐13.6
	Piercing ever but not currently	148	28	18.9	13.0‐26.2
	Currently 1 to 2 piercings	231	49	21.2	16.1‐27.1
	Currently ≥3 piercings	48	16	33.3	20.4‐48.4
61 to 74 years	Males	304	10	3.3	1.6‐6.0
	Females	308	35	11.4	8.0‐15.4
	Never piercing	362	12	3.3	1.7‐5.7
	Piercing ever but not currently	56	8	14.3	6.4‐26.2
	Currently 1 to 2 piercings	130	18	13.8	8.4‐21.0
	Currently ≥3 piercings	13	3	23.1	5.0‐53.8

### 3.5 Factors associated with nickel contact allergy

The results of a logistic regression analysis to assess different risk factors for contact allergy to nickel are shown in Table 5. Investigated variables included: sex, age (4 age groups, with the youngest as reference), atopic dermatitis during the subject's lifetime, hand dermatitis during the subject's lifetime, piercings (ever but not currently; currently 1 to 2 piercings; currently ≥3 piercings), country (with Germany, which provided the largest sample, as a reference) and body mass index (BMI) of >25. The univariate analysis showed that being female was a strong and significant risk factor for nickel allergy, with a crude OR estimate of 5.19 (95%CI: 3.99‐6.74). The risk of nickel allergy was increased in the groups aged 31 to 45 years and 46 to 60 years, and decreased in the oldest group aged 61 to 74 years. No significant association for nickel allergy and atopic dermatitis or hand dermatitis was observed. Strongly significant associations were observed between nickel allergy and ever having a piercing but not currently having a piercing (OR 3.86, 95% 2.85‐5.24), having 1 to 2 piercings currently (OR 4.00, 95%CI: 3.05‐5.24) and having ≥3 piercings currently (OR 5.58, 95%CI: 4.02‐7.76). The risk of nickel allergy was decreased in Sweden (OR 0.52, 95%CI: 0.36‐0.75) as compared with Germany, whereas a higher risk was seen for Portugal (OR 1.39, 95%CI: 1.05‐1.84). A decreased risk of nickel allergy was found in overweight females (OR 0.70, 95%CI: 0.57‐0.85).

**Table 5:** Factors associated with nickel contact allergy—logistic regression analysis

		Univariate		Multivariate	
		OR	95%CI	OR	95%CI
Sex	Male	1		1	
	Female	5.19	3.99‐6.74	3.25	2.32‐4.55
Age (y)	18 to 30	1		1	
	31 to 45	1.88	1.44‐2.46	2.15	1.60‐2.88
	46 to 60	1.39	1.06‐1.84	1.65	1.21‐2.25
	61 to 74	0.58	0.40‐0.83	0.91	0.60‐1.38
Atopic dermatitis in lifetime	No	1			
	Yes	1.17	0.79‐1.72		
Hand dermatitis in lifetime	No	1			
	Yes	1.32	0.81‐2.17		
Piercing	Never piercing	1		1	
	Piercing ever but not currently	3.86	2.85‐5.24	1.80	1.26‐2.57
	Currently 1 to 2 piercings	4.00	3.05‐5.24	1.90	1.36‐2.64
	Currently ≥3 piercings	5.58	4.02‐7.76	2.78	1.86‐4.15
Country	Germany	1		1	
	Italy	1.31	0.99‐1.74	1.19	0.88‐1.62
	The Netherlands	1.16	0.85‐1.56	1.35	0.98‐1.86
	Portugal	1.39	1.05‐1.84	1.45	1.06‐1.96
	Sweden	0.52	0.36‐0.75	0.52	0.34‐0.80
BMI	≤25	1		1	
	>25	0.70	0.57‐0.85	0.80	0.63‐1.00

BMI, body mass index; CI, confidence interval; OR, odds ratio. Multivariate logistic regression analysis including the variables sex, age, piercing, country, and BMI.

A multivariate analysis, including the variables sex, age, piercings, country, and BMI, showed that being female (OR 3.25, 95%CI: 2.15‐4.91) was still a significant risk factor for nickel allergy. In the groups aged 31 to 45 years and 46 to 60 years, there was an increased risk of nickel allergy. Having ever had a piercing but not currently having a piercing (OR 1.80, 95% 1.26‐2.57), having 1 to 2 piercings currently (OR 1.90, 95%CI: 1.36‐2.64) and having ≥3 piercings currently (OR 2.78, 95%CI: 1.86‐4.15) were significant risk factors. In Sweden, the risk of nickel allergy was still decreased, and the increase in Portugal remained significant. The risk of nickel allergy was no longer decreased in overweight females. Stratified multivariate models for males and females were analysed, and showed the following results for nickel allergy: females having ever had a piercing but not currently having a piercing (OR 1.74, 95%CI 1.15‐2.65); females having 1 to 2 piercings currently (OR 1.77, 95%CI: 1.21‐2.58); females having ≥3 piercings currently (OR 2.47, 95%CI: 1.59‐3.84); men having ever a piercing but not having a piercing currently (OR 1.70, 95% 0.72‐3.99); males having 1 to 2 piercings currently (OR 3.26, 95%CI: 1.40‐7.56); and males having ≥3 piercings currently (OR 5.59, 95%CI: 1.69‐18.52). The other risk estimates were quite comparable (data not shown).

## 4 DISCUSSION

### 4.1 Metal allergy in the general population

The present analysis provided prevalence estimates of sensitization to nickel, cobalt and chromium in the European general population. The age‐standardized prevalence of contact allergy to at least 1 metal was 15.7%, and the prevalences of contact allergy to nickel, cobalt and chromium were 14.5%, 2.1%, and 0.8%, respectively. In 2007, Thyssen et al published a review on the prevalence of contact allergy in the general population.^3^ They reported a median prevalence of nickel contact allergy based on all studies performed in the general population at that time of 8.6% (range 0.7‐27.8%). Our study shows that the prevalence of nickel allergy in the general population is high (14.5%). A European series of consecutive dermatitis patients tested in a comparable time period showed an age‐standardized and sex‐standardized proportion of nickel allergy of 22.7%, if testing with the TRUE Test was performed.^1^ The high prevalence of nickel allergy in the general population as compared with the proportion in consecutively tested dermatitis patients may suggest that individuals do not visit a doctor because of complaints related to piercings or metal objects. Regarding contact allergy to cobalt and chromium, only 1% to 3% of the general population were sensitized in previous studies.^3^ In the current study, we found similar prevalences, namely, cobalt allergy in 2.1% and chromium allergy in 0.8%.

### 4.2 Nickel allergy and regulation in different European countries

The prevalence of nickel sensitization showed wide variation among the different countries; high age‐standardized prevalences were seen in Portugal (18.5%), Italy (16.4%), The Netherlands (15.8%), and Germany (13.9%), whereas a low prevalence was seen in Sweden (8.3%). The lower prevalence in Sweden can be explained by less exposure as a result of legislation long before regulations were implemented in the other countries. In 1990, the Swedish government regulated the nickel content in ear‐piercing materials. The regulation included a ban on ear piercing with nickel‐containing piercers if the alloy contained >0.05% nickel.^9^, ^10^ In 1994, the EU Nickel Directive was approved to protect European citizens from nickel allergy, but it did not come into full force until July 2001 (1994/27/EC).^11^ Nickel was not allowed in piercings during epithelialization unless the nickel concentration was <0.05%, and nickel was not allowed in jewellery and products intended to come into direct and prolonged contact with the skin if nickel release was >0.5 μg/cm^2^/week. In 2005 the regulation was amended, and nickel was not allowed in piercings unless the nickel release was <0.2 μg/cm^2^/week from all items inserted into pierced parts of the body, not only during epithelialization after piercing (2004/96/EC).^12^ Since 2006, the nickel directive has been part of Registration, Evaluation, Authorization and Restriction of Chemical (REACH) regulation.^13^

In Sweden, the early regulation in 1990 led to a significant decrease in the proportion of consumer items that released an excessive amount of nickel.^14^, ^15^ The higher prevalence of nickel allergy in countries other than Sweden can be explained by the relatively late enforcement of the EU Nickel Directive. Insufficient implementation of the nickel regulation could possibly explain the high prevalence in Portugal.

Although the prevalence of nickel allergy has decreased since implementation of the EU nickel restriction, nickel is still a common cause of contact allergy, both in the general population and in the clinical population. This can partly be attributed to the lack of restriction regarding the many short and frequent contacts of consumers with everyday products containing nickel.^16^ Another reason for the ongoing high prevalence of nickel allergy may be the risk of nickel exposure from consumer products such as mobile phones, laptop computers, and tablet computers, as the release of nickel from these products may not comply with the regulation.^17^ In Germany, the Federal Institute for Risk Assessment reported on nickel in toys and metal construction kits for children. Overall, 41 of 168 toys exceeded the legal limit value for nickel release of 0.5 μg/cm^2^ of toy per week, and 29 of 32 metal construction kits exceeded the legal limit.^18^ Factors other than nickel regulation, such as occupational exposure, may also contribute to the ongoing high prevalence in the general population.

Interestingly, the proportion of strong or extreme patch test reactions (++/+++) varied substantially between the countries (Table 3). In Sweden, almost all allergic reactions were strong/extreme (97.6%), whereas strong/extreme reaction constituted <60% of reactions in Germany (56.9%) and The Netherlands (57.7%). The reason for the variation is not known. Perhaps the source of sensitization with regard to kinetics of nickel release from metal objects in skin contact has some significance. The frequencies of irritant and doubtful reactions also varied substantially between countries (Table 3). At least theoretically, this variation could be explained by differences in reading of patch tests, as a recent study has shown that it is difficult to discriminate between weak positive, doubtful and irritant reactions.^19^ However, arguing against this interpretation is the fact that Germany and The Netherlands had the highest share of doubtful reactions, which can be expected if the distribution of the intensities of the nickel allergy is directed towards weak reactions rather than strong ones. Furthermore, a course with participants from all testing clinics was given with live patch tested volunteers taking part, in order to calibrate the test reading before the start of the patch testing part of the present study.^5^ If we also evaluate 15 other factors of possible significance for the patch test result, this multicentre study can be classified as a study with excellent quality, as it obtains the highest scores for all factors except for lack of control of adhesiveness of the test system and for test reading only once.^20^

### 4.3 Factors associated with nickel contact allergy

The prevalence of nickel allergy increased with ever having had a piercing but not currently having a piercing, currently having 1 to 2 piercings, and currently having ≥3 piercings. This increase showed a clear dose‐response relantionship: the more piercings, the more likely sensitization to nickel. The positive correlation between the number of piercings and nickel sensitization has previously been shown in other studies.^21^, ^22^ In the multivariate model stratified for females and males, the risk of nickel allergy if the subject currently had ≥3 piercings was stronger for males (OR 5.59, 95%CI: 1.69‐18.5) than for females (OR 2.47, 95%CI 1.59‐3.84). The higher risk in males could be explained by more exposure to nickel via other jewellery than in females.

The prevalence of nickel allergy in young females (18‐30 years) was the lowest (19.8%), which may be a result of nickel regulation. The prevalence of nickel allergy in females in the middle‐aged group (31‐45 years) was high (31.8%), and the prevalence in females aged 46 to 60 years was lower (22.0%). The higher prevalence in middle‐aged females can be explained by high nickel exposure before the nickel directive was implemented. Regarding the prevalence of nickel allergy in individuals with ≥3 piercings, the prevalence of nickel allergy in the group aged 31 to 45 years was 30.4%, which was comparable with the prevalence in the group aged 46 to 60 years: 33.4%. This indicates that the prevalence of nickel allergy does not depend on the age group, but on having piercings. Although the overall prevalence of nickel allergy was somewhat lower in the oldest group aged 61 to 74 years, this was less obvious in those with ≥3 piercings (23.1%).

In the multivariate regression analysis, in the groups aged 31 to 45 years and 46 to 60 years there was an increased risk of nickel contact allergy, with the youngest age group as a reference. In the oldest group aged 61 to 74 years, there was a decreased risk of nickel allergy. The prevalence of nickel allergy decreases with increasing age, owing to different frequencies of ear piercing in different generations, and probably also because of a decrease in exposure to jewellery. It has also been reported that the immune response of the skin diminishes with ageing, owing to senescence of the immune system.^23^ This can also explain the lower prevalence of nickel allergy in the oldest age group. A recent pilot study by Lusi et al reported a higher prevalence of nickel allergy in an overweight female population.^24^ In the univariate analysis, we found the opposite; however, this effect was no longer significant in the multivariate model, and there were also no differences in effects between males and females.

### 4.4 Chromium and cobalt allergy

In the current study, the prevalence of chromium allergy was lowest (0.2%) in Sweden, in both males and females. In Sweden, ferrous sulfate has been added to cement in order to reduce the water‐soluble chromate content since 1983. In 1989, Swedish legislation came into force, stating that the chromate concentration in cement was not allowed to be >2 ppm. In 2005, the EU Directive (2003/53/EC) came into effect, and included the aforementioned limit for chromate in cement. The early legislation on the chromate concentration in cement in Sweden explains the low prevalence in males, because of a reduction in the prevalence of chromate allergy in construction workers.^25^ A high prevalence of chromium allergy in males in Portugal (1.7%) may be explained by late implementation of the regulation on chromate in cement.

Leather products have been described as important causes of chromium contact allergy in the clinical population.^26^ Leather shoes were the most frequent sources of relevant exposure in patients with chromate allergy, more so in females than in males.^27^ Leather glove exposure occurred more often in males than in females. The use of chromium in leather tanning could be a contributory factor to the high prevalence of chromium allergy in Portugal, as the prevalence in females was also high (1.0%), and people may wear shoes without socks because of the warmer weather in southern Europe. In Germany, the prevalence in both males (1.2%) and females (1.1%) was high as compared with that in other northern European countries, which can be explained by chromium in leather. The German Federal Institute for Risk Assessment reported on the chromium content of leather goods, such as gloves, shoes, and leather watch straps. More than half of the investigated samples contained hexavalent chromium, and one sixth of the samples contained more than 10 mg chromium/kg leather (http://www.bfr.bund.de/cd/9575). The release of hexavalent chromium from leather in consumer and occupational products has been limited to <3 mg/kg in the EU since May 2015 (EU 301/2014 amending annex XVII of EG 1907/2006 [REACH]).

The prevalence of cobalt allergy in the general population was higher in females (3.0%) than in males (1.1%), which can possibly be explained by exposure to cobalt in jewellery. In a clinical population, pronounced concomitant reactivity between nickel and cobalt was observed, especially in females.^1^ Other sources of cobalt exposure are other metal consumer objects, prosthetics, paints, and pigments. Concerning cobalt, there is no legislation yet to limit the amount of cobalt in consumer products.

### 4.5 Limitations

The response rates might constitute a study limitation. Selection bias at the first stage of recruitment cannot be ruled out, owing to the response rates, and might have been a reason for some of the international differences observed.

## 5 CONCLUSION

The data presented show that the prevalence of metal contact allergy in the general population was high, mostly because of nickel. The ongoing high prevalence of nickel allergy shows the importance of complying with the regulation, also including new consumer products. The lowest prevalences of both nickel and chromium allergy in Sweden support the effectiveness of long‐standing regulation.

## References

[1] Uter W , Larese Filon F , Rui F , et al. ESSCA results with nickel, cobalt and chromium, 2009‐2012. Contact Dermatitis. 2016;75:117‐121. 27385522 10.1111/cod.12582

[2] Garg S , Thyssen JP , Uter W , et al. Nickel allergy following European Union regulation in Denmark, Germany, Italy and the UK. Br J Dermatol. 2013;169:854‐858. 23909687 10.1111/bjd.12556

[3] Thyssen JP , Linneberg A , Menné T , Johansen JD . The epidemiology of contact allergy in the general population—prevalence and main findings. Contact Dermatitis. 2007;57:287‐299. 17937743 10.1111/j.1600-0536.2007.01220.x

[4] Naldi L , Cazzaniga S , Gonçalo M , et al. Prevalence of self‐reported skin complaints and avoidance of common daily life consumer products in selected European regions. JAMA Dermatol. 2014;150:154‐163. 24369385 10.1001/jamadermatol.2013.7932

[5] Rossi M , Coenraads PJ , Diepgen T , et al. Design and feasibility of an international study assessing the prevalence of contact allergy to fragrances in the general population: the European Dermato‐Epidemiology Network Fragrance Study. Dermatology. 2010;221:267‐275. 20881361 10.1159/000319757

[6] Johansen JD , Aalto‐Korte K , Agner T , et al. European Society of Contact Dermatitis guideline for diagnostic patch testing—recommendations on best practice. Contact Dermatitis. 2015;73:195‐221. 26179009 10.1111/cod.12432

[7] Ahmad OB , Boschi‐Pinto C , Lopez AD , et al. Age Standardization of Rates: A New WHO Standard. Vol 31 Geneva, Switzerland: World Health Organization; 2001.

[8] Diepgen TL , Ofenloch RF , Bruze M , et al. Prevalence of contact allergy in the general population in different European regions. Br J Dermatol. 2016;174:319‐329. 26370659 10.1111/bjd.14167

[9] General advice regarding ear piercing. National Board of Health and Welfare, Sweden, SOSFS. 1989;40.

[10] Lidén C . Nickel in jewellery and associated products. Contact Dermatitis. 1992;26:73‐75. 1633708 10.1111/j.1600-0536.1992.tb00887.x

[11] European Communities . European Dir. 94/27/EC of 30 June 1994 amending for the 12th time Dir. 76/769/EEC on the approximation of the laws, regulations and administrative provisions of the member states relating to restrictions on the marketing and use of dangerous substances*. Official J Eur Commun.* 1994;37:1‐2.

[12] Commission Directive 2004/96/EC of 27 September 2004 amending Council Directive 76/769/EEC as regards restrictions on the marketing and use of nickel for piercing post assemblies for the purpose of adapting its Annex I to technical progress. *Official J Eur Union.* 2004;L301/51.

[13] Commission regulation (EC)No. 552/2009 of June 2009 amending regulation (EC) No. 1907/2006 of the European Parliament and of the Council on the Registration, Evaluation, Authorisation and Restriction of Chemicals (REACH) as regards Annex XVII. *Official J Eur Union.* 2009;L64:7‐31.

[14] Lidén C , Johnsson S . Nickel on the Swedish market before the Nickel Directive. Contact Dermatitis. 2001;44:7‐12. 11156037 10.1034/j.1600-0536.2001.440102.x

[15] Lidén C , Norberg K . Nickel on the Swedish market. Follow‐up after implementation of the Nickel Directive. Contact Dermatitis. 2005;52:29‐35. 15701127 10.1111/j.0105-1873.2005.00494.x

[16] Erfani B , Lidén C , Midander K . Short and frequent skin contact with nickel. Contact Dermatitis. 2015;73:222‐230. 26086991 10.1111/cod.12426

[17] Jensen P , Menné T , Thyssen JP . Education, communication, and nickel allergy—now is the time for stricter discipline. Contact Dermatitis. 2013;68:116. 23289881 10.1111/cod.12030

[18] Bundesinstitut fur Risikobewertung, Germany. Press release. http://www.bfr.bund.de/en/press_information/2013/30/high_nickel_release_from_metal_construction_kits_can_trigger_allergies-188519.html. 2017. Accessed January 26, 2018.

[19] Svedman C , Isaksson M , Björk J , Mowitz M , Bruze M . “Calibration” of our patch test reading technique is necessary. Contact Dermatitis. 2012;66:180‐187. 22404193 10.1111/j.1600-0536.2011.02044.x

[20] Bruze M . Thoughts on how to improve the quality of multicentre patch test studies. Contact Dermatitis. 2016;74:168‐174. 26899806 10.1111/cod.12507

[21] Smith‐Sivertsen T , Dotterud LK , Lund E . Nickel allergy and its relationship with local nickel pollution, ear piercing, and atopic dermatitis: a population‐based study from Norway. J Am Acad Dermatol. 1999;40:726‐735. 10321601 10.1016/s0190-9622(99)70154-4

[22] Fors R , Stenberg B , Stenlund H , Persson M . Nickel allergy in relation to piercing and orthodontic appliances—a population study. Contact Dermatitis. 2012;67:342‐350. 22631615 10.1111/j.1600-0536.2012.02097.x

[23] Kwangsukstith C , Maibach HI . Effect of age and sex on the induction and elicitation of allergic contact dermatitis. Contact Dermatitis. 1995;33:289‐298. 8565482 10.1111/j.1600-0536.1995.tb02041.x

[24] Lusi EA , Di Ciommo VM , Patrissi T , Guarascio P . High prevalence of nickel allergy in an overweight female population: a pilot observational analysis. PLoS One. 2015;10:e0123265. 10.1371/journal.pone.0123265 PMC4379055 25822975

[25] Lejding T , Mowitz M, Isaksson M, et al. A retrospective investigation of hexavalent chromiumallergy in southern Sweden. Contact Dermatitis, accepted for publication. 10.1111/cod.12969 29572843

[26] Carøe C , Andersen KE , Thyssen JP , Mortz CG . Fluctuations in the prevalence of chromate allergy in Denmark and exposure to chrome‐tanned leather. Contact Dermatitis. 2010;63:340‐346. 21039593 10.1111/j.1600-0536.2010.01798.x

[27] Teixeira V , Coutinho I , Gonçalo M . Allergic contact dermatitis to metals over a 20‐year period in the Centre of Portugal: evaluation of the effects of the European directives. Acta Med Port. 2014;27:295‐303. 25017340

